# Authors’ Reply to: Critique of “Physical Activity Assessment Between Consumer- and Research-Grade Accelerometers: A Comparative Study in Free-Living Conditions” – Does Location of the Device Matter?

**DOI:** 10.2196/mhealth.7413

**Published:** 2017-02-27

**Authors:** Gregory M Dominick, Kyle N Winfree, Ryan T Pohlig, Mia A Papas

**Affiliations:** ^1^ University of Delaware Department of Behavioral Health and Nutrition University of Delaware Newark, DE United States; ^2^ Northern Arizona University Informatics and Computing Program Northern Arizona University Flagstaff, AZ United States; ^3^ University of Delaware Biostatistics Core Facility University of Delaware Newark, DE United States

**Keywords:** Fitbit, activity tracker, actigraphy, physical activity, aerobic exercise, validity

My co-authors and I thank Dr. Migueles and colleagues for their letter to the editor [1] regarding our recent *JMIR mHealth and UHealth* manuscript [2]. We welcome the opportunity to address the matters raised. 

In regard to the core critique that divergent placement of the ActiGraph and Fitbit devices (hip and wrist, respectively) confounds data interpretation in our investigation is limited, given that the methods and subsequent data interpretation were informed by the literature available at the time the study was conducted in 2014. Whereas the ActiGraph GT3X device can be worn on the wrist, the algorithms and cut-point thresholds currently available in the ActiLife software are valid only when the device is worn at the hip. Our study examined the measurement congruence between the first-available, wrist-worn Fitbit device (Flex) and the “gold standard,” waist-worn ActiGraph GT3X, in which we employed a longer assessment period (14 days) within free-living conditions that included average day- and minute-level activity, and which also comprised a range of self-reported bouts of exercise. Because our study used ActiGraph as the criterion measure for device comparison, it would have been methodologically inappropriate to place the device on the wrist. 

Both ActiGraph and Fitbit provide a proxy for the actual movements and activities of the subject as they occur in the natural environment.  Given that the hip-worn ActiGraph algorithms and cut points are benchmarked against direct clinical observations, [3,4] our study examined the ability of the wrist-worn Fitbit Flex to assess physical activity as compared to the validated estimates provided by ActiGraph within free-living conditions. Hence, this line of research continues to be tethered to evaluations that are akin to comparing “apples to oranges” in the generalized case.

Indeed, research has recently begun to utilize raw acceleration signals for developing improved algorithms for hip and wrist-worn accelerometers [5-7] that also include ActiGraph devices [8,9]. Case in point, population-based health surveillance systems such as the National Health Examination Survey (NHANES) are using raw acceleration signals to process activity data [10]. However, to date there is no consensus, regarding the use of raw acceleration signals to quantify activity or how to explicitly process data from raw signals [9]. Furthermore, it is not currently possible to access raw acceleration data from the Fitbit device; researchers must rely on activity counts determined by the proprietary algorithms used by Fitbit. Thus, the comparison of apples to oranges remains unavoidable at this point in time and as such there is robust empirical precedent for our study design [11-14]. 

Our study utilized a collective methodological approach founded on end-user practicality, but evolving toward a more scientifically appropriate means of comparison within truly free-living conditions. With this understanding, and more recent evidence supporting the use of raw acceleration signals [6,8-10], we were forthcoming in the manuscript when we highlighted the limitations of our study. Accordingly, we recommended that future studies use accelerometers that are placed on a common location. Yet, this will ultimately require some standardized process for determining what wrist-worn accelerometer algorithms to use as well as, identifying approaches to access raw acceleration signals from Fitbit. Other approaches currently being examined by our group include modeling the physical activity measures of the ActiGraph GT3X using Fitbit-derived measures of intensity, steps, and calories, and analyzing the implications of how modeling impacts bout assessment differences between the devices.

To summarize the discussion points, off-the-shelf- and research-grade physical activity monitor use continues to evolve. Our original study design, at the heart of the current discussion, was congruent with then modern scientific methods balanced with end user practicality. As such, our study serves as a research foundation to inform future research directions rather than maintain the status-quo. Our group, and presumably Migueles et al are, are part of a research collective working to better understand and quantify physical activity in a self-correcting fashion that emergent science has always followed and we value the perspective of those who share this vision. By having these discussions, we will collectively move this science forward.
